# Draft genome sequences of *Corallococcus* strains KH5-1 and NO1, isolated from activated sludge

**DOI:** 10.1128/mra.01104-23

**Published:** 2024-01-11

**Authors:** Shun Tomita, Ryosuke Nakai, Kyohei Kuroda, Hazuki Kurashita, Masashi Hatamoto, Takashi Yamaguchi, Takashi Narihiro

**Affiliations:** 1 Bioproduction Research Institute, National Institute of Advanced Industrial Science and Technology, Sapporo, Hokkaido, Japan; 2 Department of Science of Technology Innovation, Nagaoka University of Technology, Nagaoka, Niigata, Japan; 3 Department of Civil and Environmental Engineering, Nagaoka University of Technology, Nagaoka, Niigata, Japan; University of Southern California, USA

**Keywords:** myxobacteria, *Corallococcus*, draft genome, activated sludge

## Abstract

Myxobacteria are known as prolific producers of secondary metabolites with a unique and wide spectrum of bioactivities. Here, we report draft genome sequences of KH5-1 and NO1, myxobacteria isolated from activated sludge, which consist of 9.89 and 9.86 Mb, both of which have G + C contents of 70.7%.

## ANNOUNCEMENT

Myxobacteria are known as predatory bacteria, which produce various lytic enzymes and antibiotics to kill diverse organisms (1). Recently, genome mining of metagenomic data has revealed that the phylum Myxococcota is preferentially abundant in activated sludge and has tremendous secondary metabolite potential compared with other phyla (2–4). Herein, we successfully isolated strains KH5-1 and NO1, belonging to the genus *Corallococcus*, as Myxococcota isolated from activated sludge. To generate additional resources for elucidating the potential of producing novel and valuable secondary metabolites, the draft genomes of strains KH5-1 and NO1 were sequenced.

The microbial samples were collected from activated sludge tanks treating municipal sewage in the Kyushu (KH5-1) and Chubu (NO1) regions in Japan. The sludge samples (500 µL) were centrifuged at 8,000 × g for 5 min at room temperature, and then the supernatants were discarded to obtain the pellet. The strains KH5-1 and NO1 were isolated by the traditional baiting method using *Escherichia coli* K12 as prey on water agar (0.1% CaCl_2_·2H_2_O, 20 mM HEPES, 1.5% agar) supplemented with 25 µg/mL cycloheximide (5). Briefly, small portions of the sludge pellet were placed adjacent to the *E. coli* spot, and swarming predatory colonies or fruiting bodies were observed after incubation at 30°C. Then, the furthest swarm colony edge was picked up and repeatedly transferred onto VY/2 agar (0.5% dried baker’s yeast, 0.1% CaCl_2_·2H_2_O, 1.5% agar) until no other bacterial and fungal taxa were glowing up checked by the stereo microscope. For genome sequencing, the strains KH5-1 and NO1 were cultured on VY/2 agar at 30°C for 7 days. The genomic DNA of strains was extracted using a Nucleo Spin Microbial DNA kit (Macherey-Nagel, Germany) following the manufacturer’s instructions. Whole-genome shotgun sequencing was conducted using the DNBSEQ-G400R platform (MGI Tech, Shenzhen, China) at the Bioengineering Lab. Co., Ltd. (Sagamihara, Japan). A short-read library was prepared using fragmented DNA and an MGI Easy Universal DNA Library Prep Set (MGI Tech). Circularized DNA was then prepared using an MGI Easy Circularizing Kit (MGI Tech). DNBSEQ paired-end sequences (2 × 200 bp) were determined using a DNBSEQ-G400RS High-throughput Sequencing Kit (MGI Tech) and the DNBSEQ-G400 platform. The adapter sequences were trimmed using Trimmomatic v0.33 (6) (quality score cutoff, 20). We constructed and sequenced paired-end libraries on DNBSEQ-G400R. Assembly of output reads was performed using SPAdes software version 3.12.0 1 (7) (--careful, -k 21,33,55,77,99,127, --cov-cutoff auto). Assembly metrics were assessed with the DDBJ Fast Annotation and Submission Tool (DFAST) pipeline version 1.6.0 (8). The completeness and contamination were assessed using CheckM version 1.2.2 (9). Default parameters were used for all software unless otherwise specified followed by mentioning parameters where relevant. See Table 1 for additional assembly and sequencing information (Table 1). The 16S rRNA gene sequences of KH5-1 and NO1 from the genome shared the highest similarity of 99.77 and 99.84 to *Corallococcus exercitus* AB043A^T^ in the EzBioCloud database (10).

**TABLE 1:** Information for KH5-1 and NO1

Parameters	KH5-1	NO1
Genome assembly accession	BTTW00000000.1	BTTX00000000.1
SRA (Sequence Read Archive) accession	DRA017338	DRA017339
Total no. of reads	3,583,305	3,567,427
Genome size (bp)	9,892,490	9,858,692
No. of contigs	140	105
Mean coverage (×)	145	145
G + C content (%)	70.7	70.7
N50 (bp)	817,692	1,317,208
No. of CDSs	7866	7822
Completeness (%)	99.86	99.86
Contamination (%)	2.26	1.69

The genomic data of KH5-1 and NO1 will be helpful in exploring novel biosynthesis-related gene clusters to produce valuable secondary metabolites.

## Data Availability

The draft genome sequence has been deposited at DDBJ/GenBank/EMBL under the accession numbers BTTW00000000.1 for KH5-1 and BTTX00000000.1 for NO1. Raw read sequences are available in NCBI under the Sequence Read Archive accession numbers DRA017338 for KH5-1 and DRA017339 for NO1.
